# Autologous neutralizing antibody responses after antiretroviral therapy in acute and early HIV-1

**DOI:** 10.1172/JCI176673

**Published:** 2024-04-23

**Authors:** Gregory D. Whitehill, Jaimy Joy, Francesco E. Marino, Ryan Krause, Suvadip Mallick, Hunter Courtney, Kyewon Park, John Carey, Rebecca Hoh, Heather Hartig, Vivian Pae, Sannidhi Sarvadhavabhatla, Sophia Donaire, Steven G. Deeks, Rebecca M. Lynch, Sulggi A. Lee, Katharine J. Bar

**Affiliations:** 1 Department of Medicine, Division of Infectious Disease, and; 2 Center for AIDS Research, Virus and Reservoirs Technology Core, University of Pennsylvania, Philadelphia, Pennsylvania, USA.; 3 Department of Medicine, Division of HIV, Infectious Diseases & Global Medicine, UCSF, San Francisco, California, USA.; 4 Department of Microbiology, Immunology, and Tropical Medicine, School of Medicine and Health Sciences, George Washington University, Washington, DC, USA.

**Keywords:** AIDS/HIV, AIDS vaccine, Adaptive immunity

## Abstract

**BACKGROUND:**

Early antiretroviral therapy initiation (ARTi) in HIV-1 restricts reservoir size and diversity while preserving immune function, potentially improving opportunities for immunotherapeutic cure strategies. For antibody-based cure approaches, the development of autologous neutralizing antibodies (anAbs) after acute/early ARTi is relevant but is poorly understood.

**METHODS:**

We characterized antibody responses in a cohort of 23 participants following ARTi in acute HIV (<60 days after acquisition) and early HIV (60–128 days after acquisition).

**RESULTS:**

Plasma virus sequences at the time of ARTi revealed evidence of escape from anAbs after early, but not acute, ARTi. HIV-1 envelopes representing the transmitted/founder virus(es) (acute ARTi) or escape variants (early ARTi) were tested for sensitivity to longitudinal plasma IgG. After acute ARTi, no anAb responses developed over months to years of suppressive ART. In 2 of the 3 acute ARTi participants who experienced viremia after ARTi, however, anAbs arose shortly thereafter. After early ARTi, anAbs targeting those early variants developed between 12 and 42 weeks of ART and continued to increase in breadth and potency thereafter.

**CONCLUSION:**

Results indicate a threshold of virus replication (~60 days) required to induce anAbs, after which they continue to expand on suppressive ART to better target the range of reservoir variants.

**TRIAL REGISTRATION:**

ClinicalTrials.gov NCT02656511.

**FUNDING:**

NIH grants U01AI169767, R01AI162646, UM1AI164570, UM1AI164560, U19AI096109, K23GM112526, T32AI118684, P30AI045008, P30AI027763, R24AI067039; Gilead Sciences grant INUS2361354; Viiv Healthcare grant A126326.

## Abstract 2

Following antiretroviral therapy initiation in early HIV, neutralizing antibody development requires a threshold of virus replication, after which they continue to mature during antiretroviral therapy.

## Introduction

HIV-1 cure strategies aim to eradicate the proviral reservoir and/or enhance immune-mediated virus suppression. Thus, understanding HIV-specific cellular and humoral immunity is central to the cure research agenda (1). Early antiretroviral therapy initiation (ARTi) has been shown to restrict reservoir size and diversity (2–7) while preserving immune function (8), providing an optimal setting for immunologic cure interventions. Furthermore, immediate ARTi at time of HIV-1 diagnosis limits transmission (9, 10) and disease progression (11, 12) in people with HIV. This early viral suppression, however, may also abrogate development of autologous neutralizing antibodies (anAbs). Neutralizing antibody (nAb) responses against HIV develop slowly, often arising after 3 or more months of persistent virus replication (13). Other adaptive responses, such as HIV-specific cytotoxic T lymphocytes (CTLs) (14) and non-neutralizing antibodies (used in diagnostic testing algorithms and potentially mediating antibody-dependent cellular virus inhibition) (15), arise during the first 2–4 weeks of acute disease. The kinetics of CTL (16) and effector antibody responses (17) after early ARTi have been described recently, but less is known about the timing, magnitude, and durability of host nAb development in this setting. Here, we aimed to characterize the kinetics and determinants of anAb development following acute and early HIV acquisition and immediate ARTi in the UCSF Treat Acute HIV cohort (18).

Delayed development of anAbs distinguishes HIV from other viral infections, including SARS-CoV-2 (19), influenza (20), and herpesviruses (21), against which potent nAbs develop within days of disease onset. Anti–HIV-1 nAbs are classically defined as those that bind the functional HIV-1 envelope (Env) trimer and prevent infection of new target cells by sterically blocking access to cellular receptors, inhibiting necessary energy states, or impeding conformational transitions (22). HIV-1 evades effective humoral immunity and delays nAb development by impairing CD4^+^ T cell help and concealing neutralizing epitopes through dense glycosylation and conformational masking (23–26). Furthermore, the HIV virion displays many nonfunctional “decoy” antigens, such as gp41-gp120 monomers and gp41 stumps (27) with relatively few functional Env trimers (28). Most binding antibodies recognizing monomeric Env or gp41 antigens do not neutralize plasma virus (29, 30). Early anAbs are directed to target epitopes in the highly polymorphic “variable” regions of Env (31), allowing for virus escape facilitated by the high error rate of HIV-1 replication (32). As a result, the earliest anAbs lack breadth and are highly strain specific for transmitted/founder (TF) virus(es), the virus(es) that establish productive infection following a transmission event (23, 33).

Early nAb responses can be quite potent but are insufficient to control HIV replication owing to rapid immune escape. In untreated early infection, plasma virus evolves to escape activity of contemporaneous circulating anAbs as they arise (33). As untreated HIV progresses, successive cycles of viral escape from ongoing anAb responses continue, though escape may be less complete in chronic infection (34). Neutralizing antibodies with activity against a broad range of isolates can be identified in between 5%–30% of chronically infected individuals, but these broadly neutralizing antibodies (bnAbs) arise only after many cycles of antibody development and viral escape over years of viremia and are infrequently associated with virus control (35, 36). Furthermore, active HIV replication disrupts cellular immunity, inducing dysfunctional B cell (37) and T follicular helper cell (24) responses that impair effective antibody responses. Paradoxically, ongoing viral replication both provides the antigen exposure necessary for development and honing of anAb activity, and simultaneously impairs cellular immunity and allows viral escape.

Early ARTi has complex implications for anAb development. By inhibiting viral replication, ART helps preserve cellular immunity and prevents further viral evolution, but also dramatically decreases the amount of Env antigen encountered by immune cells. Halting viral replication, however, does not eliminate Env expression. Initially upon ARTi, virus is expressed by productively infected cells that exhibit a range of half-lives (38, 39). During steady-state ART suppression, HIV Env exists via expression of provirus from reservoir cells (40) or surface recycling of complement-bound virions captured within follicular dendritic cell (FDC) endosomes (41, 42). Intact virions may remain archived within FDC networks for months to years on ART (42, 43). Similarly, vaccination studies in nonhuman primate models have shown that long-lived germinal centers facilitate persistent development of nAb responses over 9 months after the last immunogen dose (44). Thus, even in the absence of viremia, sufficient antigen may be present to facilitate ongoing maturation of humoral responses.

Longitudinal cohort studies suggest that this low level of HIV antigen exposure during ART-mediated viral suppression may drive functional humoral immune responses. HIV-specific binding (i.e., non-neutralizing) antibodies appear to wane over long-term ART (45), and some participants with acute ARTi experience complete seroreversion (46, 47). Neutralizing antibodies, in contrast, appear to persist for years (48) and may even increase in potency over time (49, 50). Two recent studies of acute and early treatment cohorts inform our understanding of antibody development after early ARTi. A study of participants with acute and early ARTi (stage I through V) from the Military HIV Research Program by Mitchell and colleagues described the induction and persistence of HIV-specific binding antibodies with effector functionality following ARTi in acute HIV at stage III disease or later. Despite evidence of increased germinal center activity, however, these participants did not develop nAbs during the first year of ART (17). Esmaeilzadeh and colleagues described 12 individuals with early ARTi, some of whom demonstrated broadening of anAb titers over years of ART against autologous virus sampled at ARTi. This study also suggested the potential for those anAbs to restrict some reservoir virus populations from rebound at analytical treatment interruption (51). These reports suggest differences between anAb responses after acute versus early ARTi, as well as the potential for ongoing HIV-specific humoral immunity on suppressive ART.

Cure strategies harnessing anAbs, such as therapeutic vaccination, may differ substantially whether the desired outcome is to boost, broaden, or foster de novo anAb responses. To better inform future cure strategies, we aimed to characterize the kinetics of longitudinal HIV-specific anAb development after acute and early ARTi, rooting our assessment to autologous plasma virus populations sequenced at the time of ARTi. Our results suggest that a “threshold” of viral replication prior to, or after, ARTi is required to induce nascent anAb responses that then continue to mature during long-term suppressive ART.

## Results

### Study cohort.

Twenty-three participants from the UCSF Treat Acute HIV cohort (18) diagnosed with acute (<60 days) or early (≥60 days) HIV and initiating immediate ART were included in this study. Participant demographics are described in Tables 1 and 2. Time to ARTi ranged from 13 to 128 days after estimated date of detectable HIV infection (EDDI) (52). Median follow-up was 42 weeks (range 12–274 weeks) from date of ART initiation. Plasma from 24 weeks after ARTi was available from all but 1 participant. All participants were cisgender men of age 21–45 years (median = 28), the majority of whom (20/23, 87%) identified as men who have sex with men (MSM). The cohort was racially diverse, with participants self-identifying as Latino (7/23, 30.4%), White (5/23, 21.7%), Asian (4/23, 17.4%), Black (3/23, 13.0%), Pacific Islander (1/23, 4.3%), mixed White/Latino (2/23, 8.7%), and mixed Latino/Native American (1/23, 4.3%). All participants provided written informed consent, and the institutional review boards of UCSF and the University of Pennsylvania approved the research.

### Time to ARTi determination and participant stratification.

Time to ARTi was determined by EDDI algorithm (https://tools.incidence-estimation.org/idt/), which uses clinical testing results to estimate a date at which a viral load of 1 copy/mL would theoretically be detectable (52). Thus by “time to ARTi” we are referring to estimated time between initial productive viral replication and ARTi, and not time between virus acquisition and ART initiation, as this would include the length of an unknown eclipse period ranging 1–7 days (53). Participants were stratified by pretest probability of possessing existing anAb responses at ART initiation. Those initiating ART within 60 days of EDDI were considered “acute ART initiators” (AAi; *N* = 15), and participants initiating ART between 60 and 128 days were considered “early ART initiators” (EAi; *N* = 8). This differentiates participants with fewer than 2 months of viremia prior to ARTi, in whom baseline anAbs are likely absent (AAi), and those with 2–4 months of viremia, in whom baseline anAbs may be present (EAi).

### Viral load kinetics.

**Figure 1:**
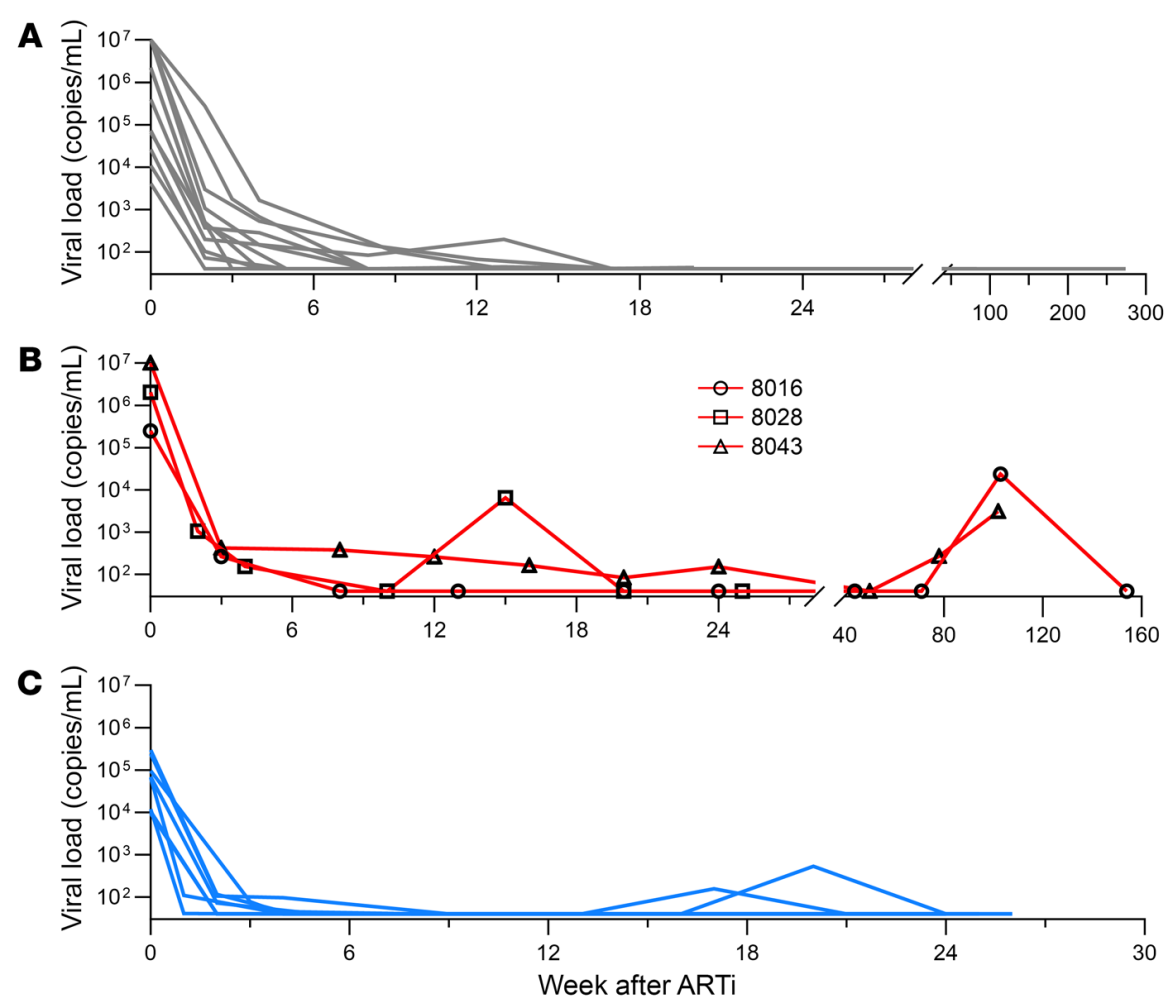
Viral load kinetics. Plasma viral load measurements in copies/mL starting at day of ART initiation (week 0) and longitudinally on ART for acute ART initiators without rebound (gray, *N* = 12) (**A**), acute ART initiators with rebound (red, *N* = 3) (**B**), and early ART initiators (blue, *N* = 8) (**C**) as measured by commercial clinical assays (limit of detection >40 copies/mL).

After ARTi, plasma virus undergoes multiphasic decay resulting from graded attrition of infected cells with variable half-lives (38, 39). ARTi at the earliest stages of disease is associated with longer times to virus suppression under clinical assay detection limits, possibly due to greater magnitude of cell infection during peak viremia of acute HIV (54). In our cohort, viral loads at ARTi were significantly higher in the AAi group (4,142 to >10,000,000 copies/mL, median of 2,026,349 copies/mL) compared with the EAi group (9,525 to 297,362 copies/mL, median of 66,503 copies/mL) (*P* = 0.02, 2-tailed Mann-Whitney). Accordingly, first undetectable viral load (<40 copies/mL) was observed between 2 and 50 weeks after ARTi (median of 5 weeks) and trended later in AAi (median = 8 weeks) than in EAi (median = 4 weeks) (*P* = 0.08, 2-tailed Mann-Whitney) (Tables 1 and 2 and Figure 1).

Persistent or intermittent low levels of detectable viremia after ARTi occur via several mechanisms, including incomplete drug adherence enabling ongoing viral replication, full suppression of viral replication with continued virus production from longer-lived infected cells, and stochastic expression of provirus from activated reservoir cells (40). Whether this low-level viremia on ART can contribute to anAb development is unknown, but we noted several participants with detectable viremia after 24 weeks of ART. Participant 8043 initiated ART during acute HIV, and subsequently experienced a prolonged time to viral suppression. Following rapid reduction in viremia from >10,000,000 to 422 copies/mL in the first 4 weeks on ART, participant 8043 then maintained low-level viremia through 50 weeks of ART (Supplemental Figure 1; supplemental material available online with this article; https://doi.org/10.1172/JCI176673DS1). After achieving virus suppression on ART, a total of 7 participants demonstrated a subsequent detectable viral load (>40 copies/mL by commercial assay; Tables 1 and 2 and Supplemental Figure 1). Three of these participants (8016, 8028, and 8043) initiated ART during acute disease, achieved suppression, and then had an episode of measured viremia greater than 1,000 copies/mL (Figure 1B, red). No planned interruptions in therapy were guided by the participants’ clinicians, but participant report and drug level testing confirmed ART nonadherence during these rebound viremic episodes (data not shown). Virus rebound after analytical treatment interruption has been shown to boost anAb responses and elicit anAbs in those without detectable titers (34, 55); thus, we considered these 3 participants as a distinct group from those in whom AAi was followed by continuous virus suppression.

### Gp120 binding antibodies.

**Figure 2:**
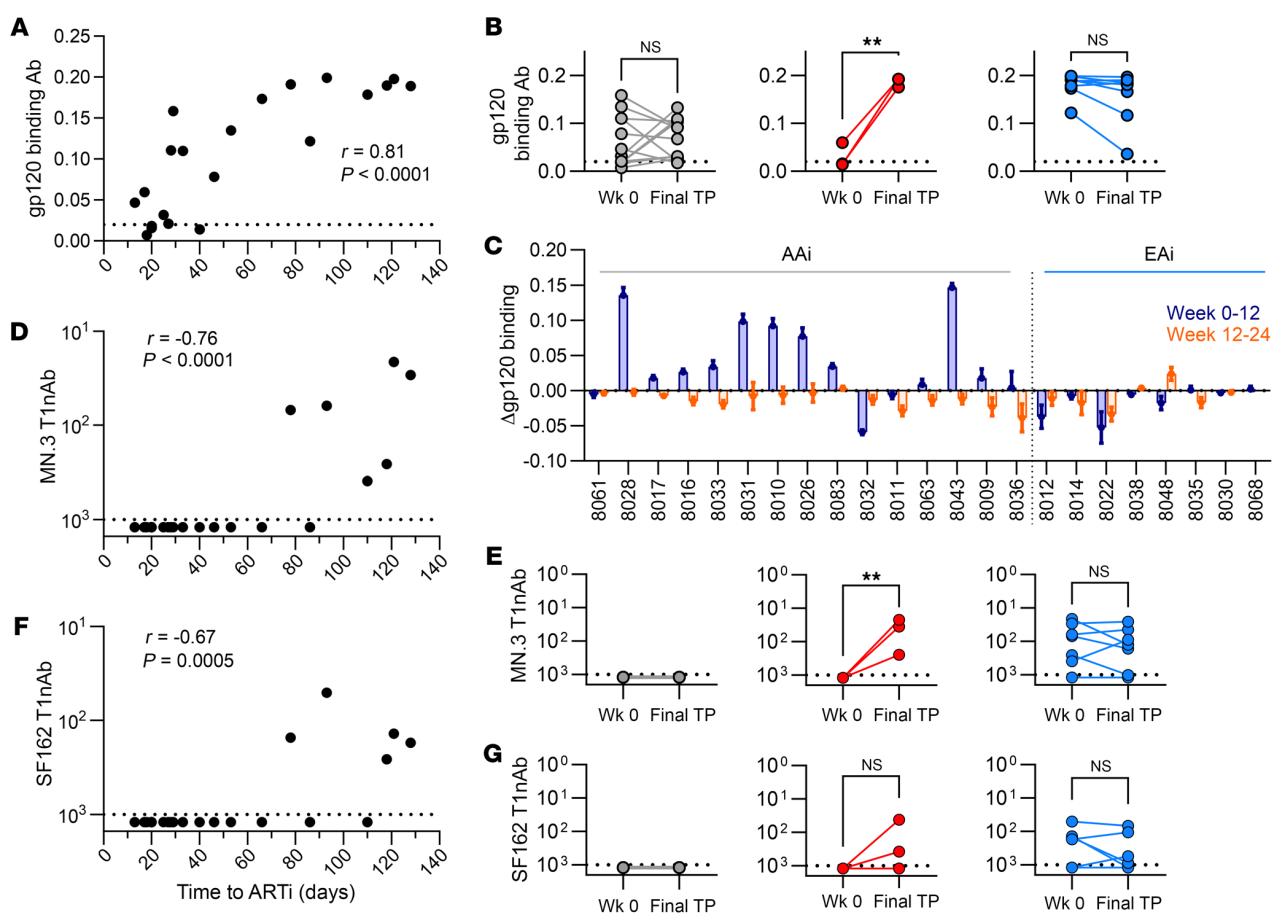
Binding and tier 1 antibody responses. (**A**–**C**) Plasma gp120 binding antibody responses as measured by qualitative ELISA and presented as area under the curve measurement. (**D**–**G**) Plasma IgG neutralization of clade B tier 1 viruses MN.3 and SF162 measured by TZM.bl assay and presented as IC_50_ in micrograms per milliliter for 23 participants. For **B**, **E**, and **G**, AAi without rebound (*N* = 12) are shown in gray, AAi with rebound (*N* = 3) in red, and EAi (*N* = 8) in blue. (**A**) Baseline gp120 binding and responses correlate with time to ART initiation (Spearman’s correlation). (**B**) Change in binding antibody responses between time of ART initiation (week 0) and final time point (Final TP; range 12–276 weeks) (Wilcoxon’s matched pairs signed-rank test). (**C**) Changes in binding antibodies over weeks 0–12 on ART (blue) compared with weeks 12–24 on ART (orange) for each participant. (**D** and **F**) Baseline tier 1 responses correlate with time to ART initiation (Spearman’s correlation). (**E** and **G**) Change in tier 1 responses on ART (Wilcoxon’s matched pairs signed-rank test). **P < 0.01.

The earliest HIV Env-specific antibody responses are non-neutralizing. Cross-reactive responses to gp41 arise in the first 2 weeks of viremia, and de novo gp120-specific binding responses are detectable within 4 weeks (30, 56–58). As they represent the earliest de novo Env-specific antibody responses, we measured gp120-specific plasma binding antibodies at ARTi in all participants. Binding antibodies were present at ARTi in 16 of 23 (70%) participants (Supplemental Figure 8), and magnitude correlated with time to ARTi (Spearman *r* = 0.81, *P* < 0.0001; Figure 2A). Participants with negative third-generation clinical ELISA testing (EDDI of <20 days) had low or no detectable baseline gp120 binding responses. In participants initiating ART between 20 and 90 days after EDDI, baseline binding antibody levels increased with longer pre-ART viremic period, until reaching a plateau in participants with viremia for 90 days or more prior to ARTi.

After ARTi, binding antibody levels did not rise significantly in either AAi or EAi participants who maintained virus suppression (Figure 2B), but responses were more dynamic in the initial weeks of HIV infection. As shown in Figure 2C, binding antibodies rose over the first 12 weeks on ART in most participants with AAi (9/15, 60%) and none with EAi (0/8, 0%) (*P* = 0.015, 2-tailed Fisher’s exact test; Figure 2C). Change in binding antibodies correlated positively with markers of acute infection, including prolonged time to first undetectable viral load (Spearman *r* = 0.424, *P* < 0.5; Supplemental Figure 2A) and higher viral load at ARTi (Spearman *r* = 0.6475, *P* < 0.001; Supplemental Figure 2B). After the initial weeks of ART, binding antibodies stabilized or decreased in 22/23 (96%) participants in the ensuing 12–24 weeks (Figure 2C). Only participant 8048, who experienced new low-level detectable viremia (528 copies/mL) at week 20, showed increased binding antibodies during this period. In AAi, only those participants with subsequent rebound showed a significant increase in binding antibodies between ARTi and the final time point (Figure 2B). Together, data suggest that binding antibody development is determined by viremia during the first 8–12 weeks of infection, and continued viremia following AAi does not further drive binding antibody responses.

### Tier 1 neutralizing antibody responses.

Tier 1 HIV viruses, such as MN and SF162, are laboratory-adapted strains that possess open, non-native Env conformations rendering them highly sensitive to neutralization by a broad range of antibodies (59, 60). Antibodies that neutralize tier 1 viruses can be elicited by monomeric gp120 protein but are not protective against HIV virus in vivo (61, 62). Distinct from binding antibodies and anAbs, emergence of tier 1 neutralizing antibodies (T1nAbs) may represent an intermediate stage in the progression of these Env-specific humoral responses, indicating de novo responses to immunogenic epitopes of the Env trimer that are well shielded in the “functional” trimeric Env of primary virus isolates. We assessed for plasma T1nAbs at ARTi and longitudinal time points.

At ARTi, T1nAbs were absent in AAi and present only in later EAi (>90 days), correlating positively with time to ARTi (MN.3 Spearman *r* = –0.76, *P* < 0.0001; SF162 Spearman *r* = –0.67, *P* = 0.0005; Figure 2, D and F). In contrast with binding antibodies, T1nAbs did not subsequently increase on suppressive ART in AAi. In the 3 participants with AAi and rebound, however, T1nAbs did develop (Figure 2, E and G). T1nAbs were detectable in the majority of EAi participants (6/8, 75%) at ARTi and did not change significantly over ART (Figure 2, E and G). Like the AAi participants, the 2 EAi participants with absent T1nAbs at ARTi, 8022 and 8012, did not later develop T1nAbs on ART.

### Sequence characterization of early virus lineages.

**Figure 3:**
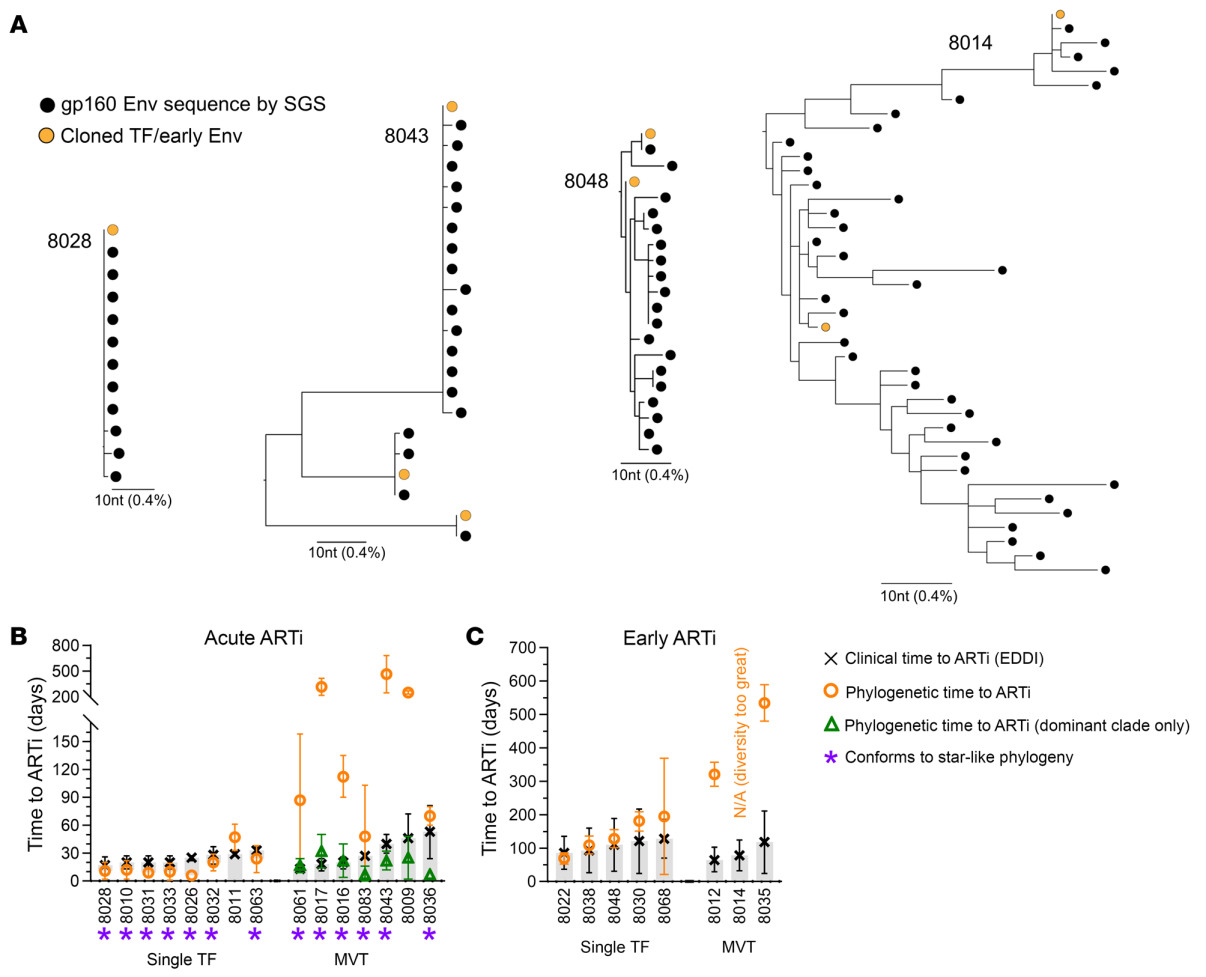
Viral populations at ART initiation. (**A**) Representative viral populations at ART initiation by SGS of gp160 env presented as maximum-likelihood nucleotide phylogenetic trees for 4 participants: 8028 represents AAi with single-virus transmission, 8043 represents AAi with multivariant transmission (MVT), 8048 represents EAi with single-virus transmission, and 8012 represents EAi with MVT. (**B** and **C**) Time to ART initiation, 95% confidence interval as estimated by clinical testing (EDDI algorithm, black/gray), and viral population diversity (Los Alamos National Laboratory Poisson Fitter tool, orange). For AAi participants with multivariant infection, diversity estimate was also performed within dominant clade only (green). Purple asterisks denote sequences conforming to star-like phylogeny.

To assess the multiplicity of infection and extent of virus evolution that had occurred prior to ARTi, we characterized plasma virus at the time of HIV diagnosis and ARTi. Single genome sequencing–derived (SGS-derived) gp160 env from plasma virus at ARTi in all 23 participants (*n* = 425 total sequences, median 18 per participant) are displayed in maximum-likelihood phylogenies (Supplemental Figures 3–6; representative examples in Figure 3A). Of the sequences generated, 40 of 425 (9.4%) contained nonsense or frameshift mutations, which were included in nucleotide phylogenies but discarded from amino acid phylogenies. Using visual inspection and a validated model of random virus evolution (63), wherein sequences conform to star-like phylogeny (SLP) in the absence of either multivariant transmission or adaptation to selective pressure, we enumerated the multiplicity of infection.

Among the AAi participants, 8 of the 15 participants’ sequences demonstrated a single, low-diversity lineage that conformed to SLP or near-SLP, indicating productive infection with a single virus (Supplemental Figure 3). Molecular clock estimates from these sequences predicting the time since infection aligned closely with clinical estimates (Figure 3B), though these estimates may be less accurate in populations not conforming to SLP (63). The other 7 AAi participants’ sequences did not conform to SLP but demonstrated 2 or more distinct low-diversity lineages suggesting acquisition of multiple related but genetically distinct TF viruses, hereafter referred to as multivariant transmission (MVT) (Supplemental Figure 4). Within these AAi MVT participants, each distinct lineage conformed to SLP or near-SLP and matched clinical timing estimates despite far greater diversity within the overall sequence alignment (Figure 3B). Lineages exhibiting SLP in these AAi participants allowed for inference of the TF env sequence as the common ancestor of that population. Inferred TF envs for 14 of 15 AAi participants were cloned to be tested phenotypically (Supplemental Figures 3 and 4). In MVT participants, the TF representing the inferred dominant clade (representing >50% of sequences) was cloned. We additionally attempted to clone minor-clade TF viruses, but some Envs lacked infectivity in functional assays; thus only dominant-clade virus was tested for participants 8061, 8017, and 8009 (Supplemental Figure 4).

In the EAi participants, sequences demonstrated greater diversity and defied SLP, suggesting virus adaptation to immune pressure prior to ARTi (Figure 3C). While CTLs can exert potent pressure on viral populations in acute infection, anAbs function as the primary selective pressure at the Env locus (32, 64, 65). Five of the 8 EAi participants had relatively low-diversity sequence populations, consistent with single virus transmission and early virus adaptation (e.g., Figure 3A, participant 8048). Given sequence evidence of virus adaptation, the TF virus could not be inferred, but 1 or more representative “early” Envs, suspected to be immune escape variants, were cloned (Supplemental Figure 5). Three EAi participants demonstrated high levels of sequence diversity suggesting MVT with subsequent diversification via selection and recombination (e.g., Figure 3A, participant 8014). In these participants, Envs representing different regions of the phylogeny were cloned for testing (Supplemental Figure 6). In total, 29 infectious pseudoviruses were generated. Across the entire cohort, MVT was identified in 10 of the 23 participants (43%).

### Autologous neutralizing antibody responses.

**Figure 4:**
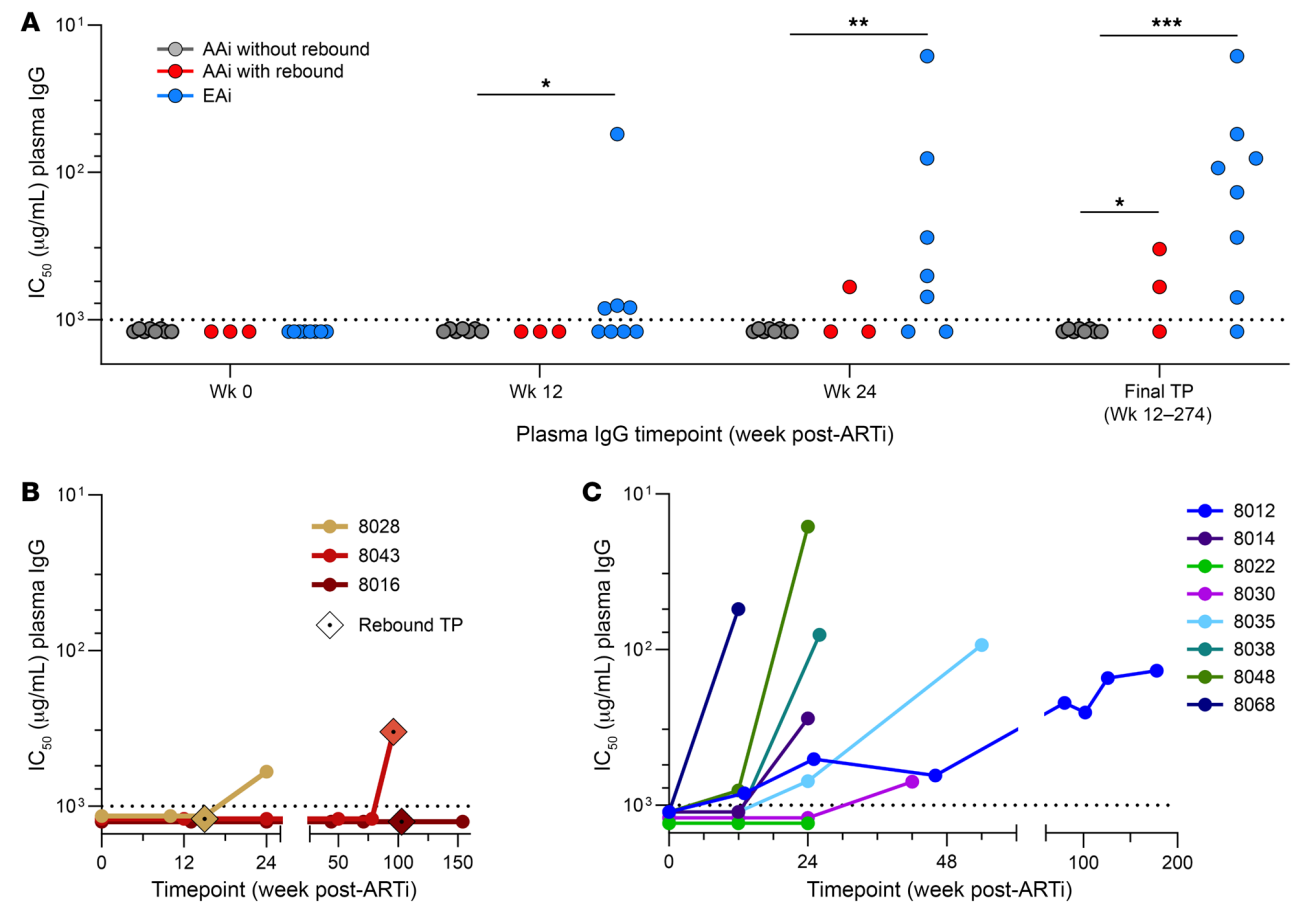
Autologous neutralizing antibody responses. Autologous neutralizing antibody (AnAb) responses of purified plasma IgG against pseudotyped autologous TF or early virus measured by TZM.bl assay and presented as IC_50_ (μg/mL) for 22 participants. AAi without rebound (*N* = 11) are shown in gray, AAi with rebound (*N* = 3) in red, and EAi (*N* = 8) in blue. (**A**) AnAb responses at ART initiation (week 0) and longitudinal time points on ART. Statistics represent response rate for relative number of participants with detectable anAbs between groups at each time point (Fisher’s exact test; *P < 0.05, **P < 0.01, ***P < 0.001). (**B** and **C**) AnAb responses over time on ART in individual acute participants with rebound (**B**) and early participants (**C**).

Using the inferred TF or early (likely anAb escape) variants, we measured anAb responses in 22 participants (Figure 4). At the time of ARTi, we expected no plasma neutralization of contemporaneous Envs, because either (a) anAbs had not yet developed, or (b) contemporaneous virus would have escaped from nascent anAbs. As expected, week 0 plasma IgG did not neutralize autologous virus in any participant (Figure 4A). After ARTi, AAi participants with continuous ART suppression (*n* = 11) failed to develop anAbs at any longitudinal time point when followed from 12 to 274 weeks (Figure 4A).

In contrast, anAbs did develop in 2 of 3 (66%) AAi participants with a rebound viremic episode after ARTi (8028 and 8043). In these 2 participants, anAbs were detected either at (participant 8043) or after (participant 8028) a rebound viremic time point (Figure 4B), though it is unclear from clinical data the duration of viremia prior to sampling.

AnAbs developed in 7 of 8 (88%) EAi participants after ARTi. AnAbs were first detected at week 12 in 4 participants (8012, 8035, 8048, 8068), week 24 in 2 participants (participants 8014, 8038), and week 42 in 1 participant (participant 8030) (Figure 4C). Notably, potency continued to rise over longitudinal time points in 4 of the 7 who developed anAbs, indicating continued evolution of the anAb response after many months or years of ART suppression. In the other 3 participants, anAbs were only detected at the last available sampled time point.

### Rebound virus populations in AAi participants.

**Figure 5:**
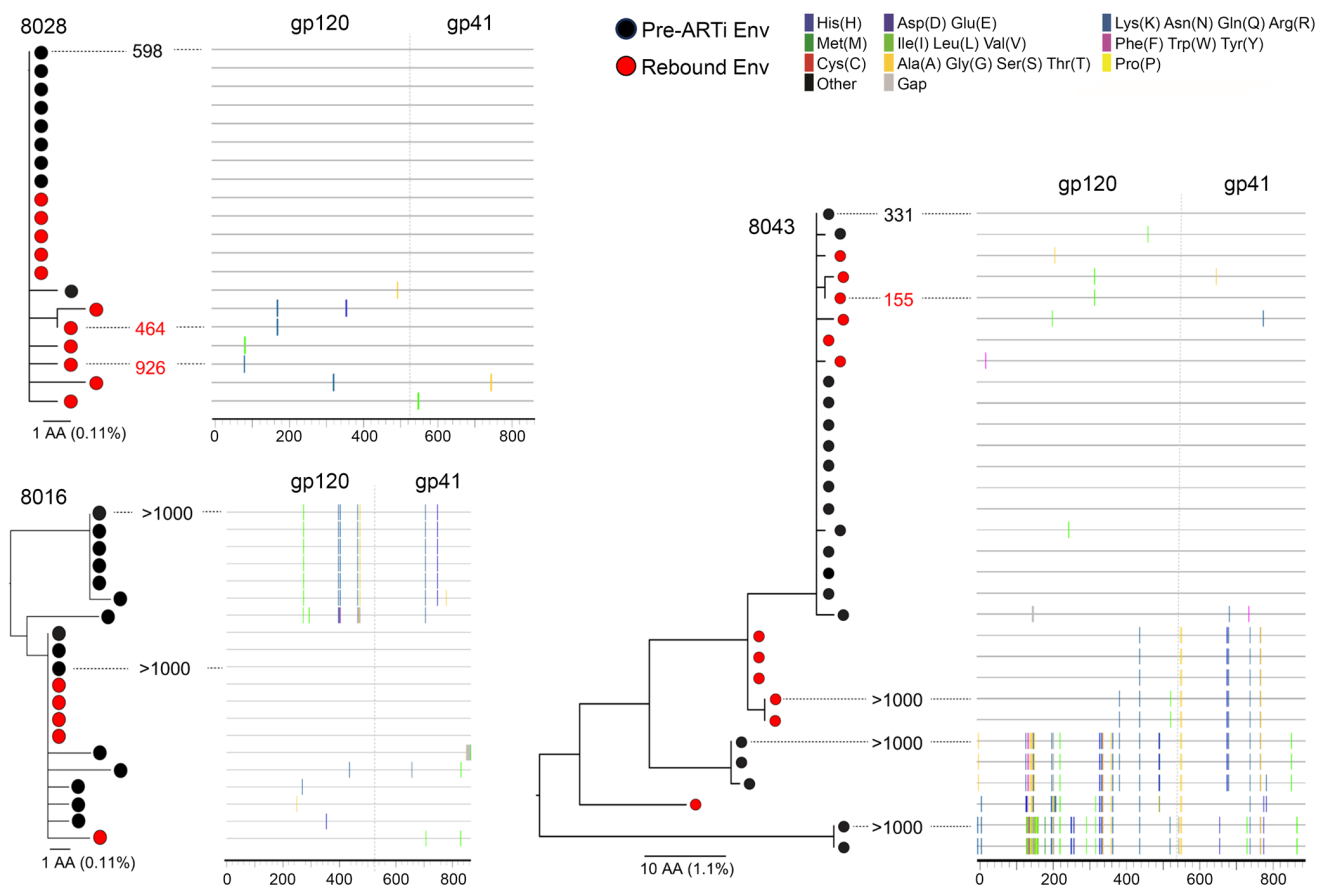
Rebound virus populations in 3 AAi participants. Maximum-likelihood amino acid phylogenetic trees and highlighter plots for the 3 AAi with rebound participants (8028, 8016, and 8043). Black nodes represent sequences obtained from plasma at ART initiation, and red nodes represent sequences obtained at rebound time point plasma. Numeric values represent final time point plasma IgG neutralization IC_50_ (μg/mL) of selected pre-ART and rebound time point Envs by TZM.bl assay.

In the 3 participants with AAi and subsequent rebound, plasma virus env sequences from rebound viremic time point were sequenced (*n* = 28, median 11 per participant) and are displayed with acute plasma virus (Figure 5). Owing to intermittent sampling, we do not know the exact timing, duration, or magnitude of rebound viremia apart from sampling time points.

Rebound was detected in participant 8016 at week 103, after more than a year of virus suppression. Rebound virus was largely identical to 1 of the 2 TF lineages identified during acute infection (Figure 5), demonstrating lack of virus evolution from acute viremia. Binding antibodies and T1nAbs against MN but not SF162 transiently increased before detection of rebound but did not continue to rise thereafter (Supplemental Figure 7A). Plasma antibodies from before, during, and after rebound failed to neutralize either rebound or TF lineages (Figure 5 and Supplemental Figure 7A). This lack of anAb development, combined with absence of sequence evolution in rebound plasma virus, suggests a limited duration of rebound viremia.

Participant 8028 had detectable rebound at week 15, shortly after the first undetectable viral load post-ARTi, and then developed low-level anAbs at week 24 (Figure 4). Rebound sequences were identical or nearly identical to the single TF virus lineage (Figure 5). No neutralizing activity was detected in rebound time point plasma IgG, though binding antibodies and T1nAbs were increased from prior measures (Supplemental Figure 7A). The minimal diversity contained in the rebound Envs included 1–2 amino acid substitutions, including 2 shared mutational motifs (sites: K178N and alteration of potential N-linked glycosylation site [PNGS] at position 88: V89I, N88K). Envs containing the K178N mutation were similarly neutralized by week 24 plasma, while the loss of the PNGS at position 88 conferred a modest increase in resistance to plasma anAbs (IC_50_ = 926 μg/mL vs. 598 μg/mL), suggesting possible early escape (Figure 5).

Participant 8043 demonstrated virus suppression at week 50 after a prolonged period of low-level viremia during the first 24 weeks of ART. At the next sampling at week 102, anAb responses neutralizing the largest of 3 TF lineages had developed (Figure 4). Rebound virus aligned with minimal diversification to this dominant lineage, as well as with 2 other lineages reflecting recombinants or previously unsampled lineages (Figure 5). The dominant clade represented a smaller proportion of the rebound population (6 of 12 sequences, 50%) compared with the pre-ARTi population (14 of 19 sequences, 74%). Within the dominant clade, 2 sequences shared an I294V substitution that did not confer resistance to anAbs when tested in vitro (IC_50_ = 155 μg/mL). Both minor lineages (sampled only pre-ARTi) and the recombinant lineage (sampled only at rebound) were resistant to anAbs (IC_50_ > 1,000 μg/mL). Three mutations in the gp120 region of this recombinant lineage differed from dominant clade TF virus: N362K in C3 region, S411N in V4, and I491V in C5. To our knowledge these mutations have not previously been described in early anAb or bnAb escape. Of the three, we note that N362K leads to PNGS loss at this site adjacent to the CD4 binding site (CD4bs) and thus may affect CD4 binding kinetics or sensitivity to neutralization by antibodies targeting CD4bs and CD4-induced epitopes (66–69). The shift in lineage frequencies suggests that anAbs may elicit pressure, but persistence of the sensitive lineage within the circulating plasma population indicates that anAbs of this titer are insufficiently potent to block replication completely.

### Autologous neutralizing antibody responses in EAi participants with MVT.

**Figure 6:**
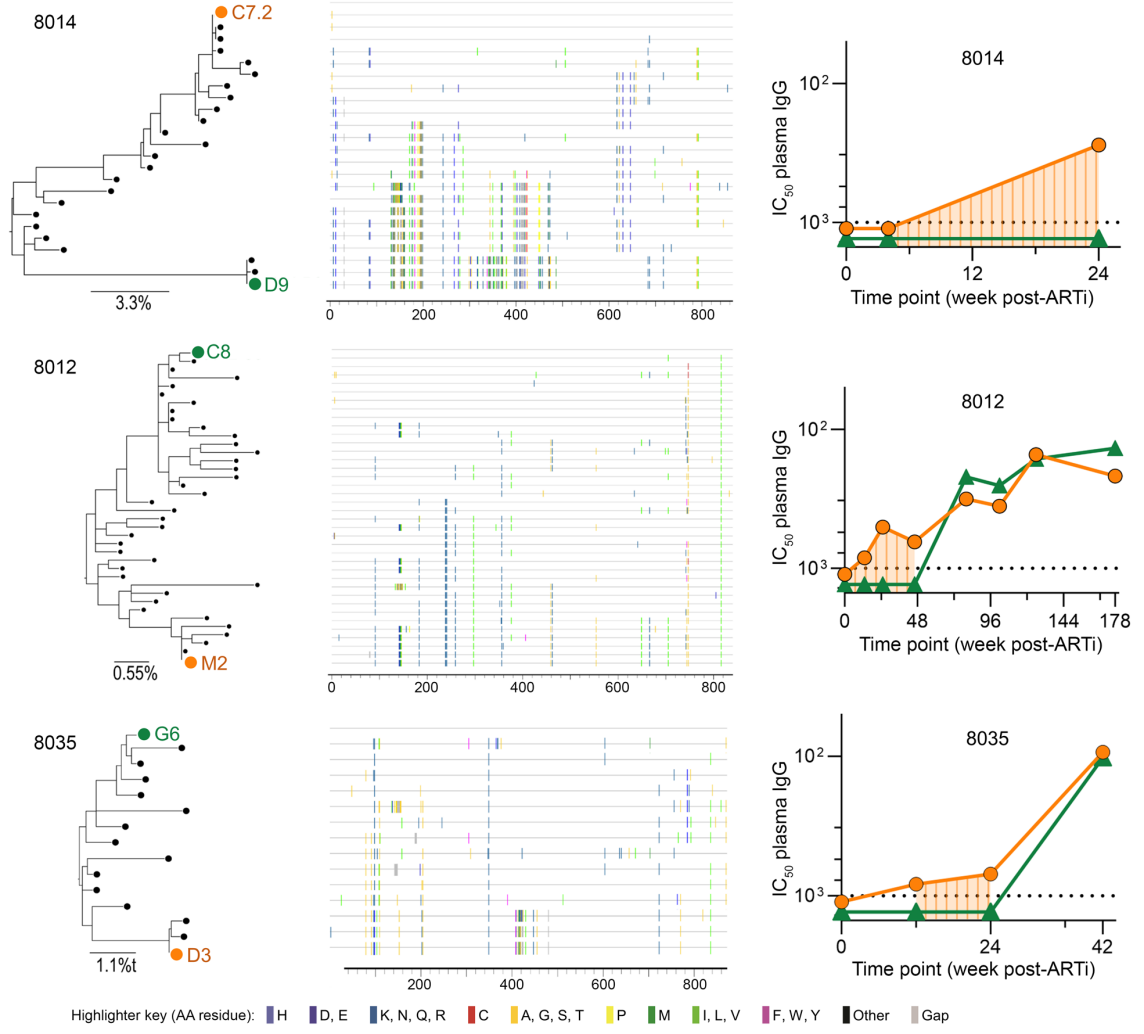
AnAb responses in EAi participants with MVT. Maximum-likelihood amino acid phylogenetic trees and highlighter plots for the 3 EAi participants with MVT are presented on the left. Pseudotyped dominant-clade and minor-clade early viruses are denoted in orange and green, respectively. Neutralization IC_50_ (μg/mL) by longitudinal plasma IgG of each clade is presented on the right. Shaded area represents time during which anAbs against only 1 variant were detected.

Three participants with EAi (8012, 8014, and 8035) and 1 with AAi and subsequent rebound (participant 8043) had MVT, affording the opportunity to assess the specificities and kinetics of anAb responses against distinct viral lineages over time (Figure 6). Notably, as the frequency of recombination events was high, determination of dominant clades (in general, >50% sequences) and minor clades (<50% of sequences) was imprecise.

Sequences from participant 8014 suggest at least 2 clades (represented by C7.2 and D9) diverging by 92 amino acids (10.2%) with multiple recombinant lineages. Despite substantial recombination, C7.2 had a larger frequency of related sequences compared with D9. In samples available through 24 weeks of ART, plasma IgG neutralized only the dominant clade. This mirrors the anAb responses that developed after rebound in participant 8043, which neutralized the dominant clade but not minor clades or recombinant variants (Figure 6).

Participant 8012’s virus population was more closely related, with 2 clades (C8 and M2) diverging by 23 amino acids (2.7%). The M2 clade was nominally more prevalent at ARTi (20/39 sequences, 51%). Neutralization of M2, but not C8, was first detected in week 12 plasma IgG and remained the only targeted lineage through 48 weeks. By week 80, however, plasma IgG neutralized both variants with comparable potency. Despite ongoing virus suppression, responses against both lineages generally increased throughout 178 weeks of monitoring (Figure 6).

Participant 8035’s virus likely represented 2 lineages with recombination and ongoing diversification, with a dominant lineage represented by sequence G6 (12/15, 80%) and a minor lineage D3 that differed by 28 amino acids (3.2%) primarily in V4–5. At 12 and 24 weeks of ART, plasma neutralized only D3. More potent responses equally targeting both lineages arose by week 42 (Figure 6).

Across the participants with MVT who developed anAbs, neutralizing responses on ART increased in potency (rising over sampled time points through 24 to 178 weeks) and breadth (targeting the one lineage first, then expanding to recognize the other). As each individual had an initial response against the TF that was present at ARTi (as indicated by escape variants in sequences at ARTi), this indicates that if anAb responses are initiated during viremia prior to ART, they continue to evolve in both potency and breadth over months to years of suppressive ART.

### Heterologous neutralizing antibody responses.

In untreated HIV infection, the earliest anAb responses are strain specific for the individual’s TF virus. Having observed that anAbs develop autologous breadth after early ARTi, we then assessed for heterologous neutralizing activity in the 7 EAi participants with detectable anAbs (8012, 8014, 8030, 8035, 8038, 8048, and 8068). Plasma IgG from the time point with the highest autologous neutralization potency was used in the TZM.bl assay against a heterologous panel of tier 2 viruses including 4 viruses from a standardized “global” panel (TRO.11, X1632, X2278, CE1176) (70), the clade B TF virus WITO (71), and BG505, an key Env in vaccination strategies. No heterologous neutralization was detected in any participant plasma IgG against any tested virus.

## Discussion

Neutralizing antibodies are the primary immune pressure driving virus escape at the HIV Env locus (65) and are increasingly recognized as a potential mechanism of virus control both in unique hosts who naturally suppress viremia (55, 72) and in the context of passive bnAb administration (73–78). Similarly, anAbs suppress reactivation of a subset of reservoir viruses in some individuals with chronic ART initiation (6, 79) and impact rebound virus populations after ART interruption in some individuals with early ART initiation (51). In the modern era of HIV cure, combination immunologic interventions will be necessary to successfully control reactivating reservoir viruses (80); nAbs may be an integral part of these strategies. Determining the natural history of the anAb response and the extent to which initial responses mature during ART may inform future efforts to augment these responses. Here, we characterized HIV-specific antibody responses in a well-characterized clinical cohort of people with HIV initiating immediate ART after diagnosis of acute and early HIV-1.

Our first finding was that participants initiating ART in acute HIV (<60 days; AAi) did not subsequently develop anAb responses during suppressive ART. No AAi participants showed sequence evidence of virus escape from anAbs, or detectable plasma neutralization, at the time of ARTi (Figures 3 and 4 and Supplemental Figures 3 and 4). This allowed for inference of TF virus(es) and indicated that initial anAb responses had yet to develop. In 11 participants who then maintained virus suppression on ART, no plasma neutralizing activity developed over many months of follow-up (Figure 4A). Tier 1 neutralizing responses were likewise absent at and after ARTi (Figure 2, E and G). Binding antibody responses, in comparison, were driven by viremia over the first 1–3 months and continued to rise on ART over the next 12 weeks (Figure 2C). Together, results show that antigen exposure following AAi drives limited evolution of humoral immunity but does not elicit detectable de novo neutralizing responses against trimeric Env even in individuals with lengthy periods of viremia after ARTi.

In contrast with AAi, participants who initiated ART during early HIV (60–128 days; EAi) developed nAb responses prior to ARTi that continued to expand in breadth and potency over time on suppressive ART. At ARTi, plasma antibodies did not neutralize contemporaneous virus, but most did neutralize tier 1 viruses (Figure 2, D and F). Also, viral sequence diversity reflected selective pressure suggesting escape from the initial anAb response (Figure 3B). Over time on ART, plasma antibodies evolved to neutralize these early escape variants in 7 of 8 individuals and further evolved to neutralize divergent variants in 2 of 3 participants with MVT (Figure 6). In parallel, anAb responses increased in potency over the duration of the follow-up period in 4 participants with available longitudinal samples, including in 1 participant followed for several years (8012; Figure 4C). Tier 2 heterologous neutralization as assessed against a panel of 6 viruses, however, did not develop in any participant.

Continued maturation of the anAb response on suppressive ART after early, but not acute, ARTi suggests a “threshold” of systemic virus replication prior to ARTi required to sufficiently induce B cell recognition of trimeric Env epitopes associated with neutralization of TF virus(es). If this threshold is not reached, then anAbs do not develop, even with several additional months of detectable viremia on ART. If this threshold is surpassed, anAbs recognizing the TF virus develop and continue to mature on ART, accruing increased breadth and potency over long periods of time without detectable viremia. Thus, substantial systemic virus replication is necessary to initiate the anAb response, but not to mature preexisting responses. Ongoing HIV replication during suppressive ART has been debated, with evidence indicating it is negligible (81–83). HIV antigen, however, is available to B cells through stochastic expression of infected reservoir cell provirus and within lymphoid tissue via captured virus in FDC endosomal networks. Our results suggest that this continued antigen presentation during ART preferentially engages with memory B cell populations to mature existing responses rather than with naive B cells to generate de novo responses, though we did not directly assess B cell biology in this study. Further elucidation of the interactions between distinct B cell subsets and different modes of antigen presentation on ART that drive continued anAb responses warrants study.

The observation of an apparent “threshold effect,” before which anAb responses do not develop and after which they arise and continue to evolve on suppressive ART, was unexpected as the immunologic consequences of earlier ART initiation would seemingly favor anAb development. Initial Env-specific responses skew toward gp41, likely from priming cross-reactive memory B cells originally targeting gut microbial antigens (57, 58). Replicating virus produces soluble monomeric gp120 and membrane-bound gp120-gp41 monomers, while trimeric Env is sparse, leading to serodominance of “binding” responses against gp120 that target non-neutralizing epitopes inaccessible on functional trimers (56). These binding antibodies may paradoxically contribute to depletion of uninfected “bystander” CD4^+^ T cells (84). Naive B cells recognizing neutralizing epitopes require antigen presentation on FDC networks and T cell help from T follicular helper (Tfh) cells to class-switch and affinity-mature (85), which may be greatly impaired by depletion of Tfh subsets over longer periods of untreated HIV. Furthermore, the intensely inflammatory milieu resulting from active viral replication interferes with optimal B cell responses, driving expansion of short-lived, activated, exhausted memory B cell populations (37, 86, 87). In the context of these known and potentially other yet undiscovered mechanisms by which HIV replication impairs effective humoral responses, it is surprising that acute ARTi does not support anAb development, and thus the mechanisms of the threshold effect, also seen in other acute ARTi cohorts (17), merit further investigation.

While no AAi participants developed anAb responses on suppressive ART, it is notable that 2 of 3 participants with documented rebound viremia produced anAbs at the time viremia was detected or shortly after. In participant 8043, neutralization-resistant variants comprised a larger percentage of the plasma virus population at the time point anAbs were detected relative to ARTi, suggesting immune selection imposed by anAb activity. The exact timing of recurrent viremia and nascent anAb emergence was not clear, but the available data suggest that rapid induction of anAbs may be possible even many months or years after AAi. Thus, while on-ART viremia of AAi may be insufficient to induce anAb responses, B cells are primed for rapid maturation following a sufficient antigen “boost” such as post-rebound virus replication or, potentially, therapeutic vaccination.

To date, therapeutic HIV vaccination strategies have largely aimed to elicit T cell responses owing to the established correlates of CTL activity with spontaneous virus control; only recently has testing of immunogens targeting B cell lineages been proposed in people with HIV. In contrast, preventive vaccine efforts have long aimed to elicit broadly reactive nAbs, a formidable challenge. Current approaches to cultivate bnAbs involve targeting rare B cell lineages, then guiding stepwise B cell maturation through a series of improbable mutations to train and polish these responses (88). Our findings demonstrate that the period of viremia preceding acute and early ARTi provides a potent and enduring antigen “prime,” which may be a reasonable target for continued stimulation via vaccination. The EAi MVT participants described here further demonstrate a natural broadening of anAb responses after early ARTi, which may represent development of new B cell lineages or continued evolution of an initial B cell lineage. Thus, therapeutic vaccination with “boosting” immunogens represents a distinct and potentially feasible approach to mature and broaden preexisting anAb responses.

Reservoir diversity following chronic ART initiation is vast, and the full complement of potential rebound virus populations cannot be reliably predicted by reservoir or pre-ART plasma virus sequencing (89). Acute and early ARTi, however, substantially restricts reservoir size and diversity. Plasma sampling at the time of early ARTi allows for more robust prediction of reservoir variants and thus assessment of reservoir sensitivity to antibody responses (78, 90). Given the advances in HIV vaccinology, including mRNA–lipid nanoparticle technologies and prefusion-stabilized trimer immunogens that have successfully elicited autologous tier 2 nAb responses in HIV-naive individuals (91), therapeutic vaccination to enhance baseline anAb responses after acute/early ARTi represents an intriguing and potentially feasible approach that merits consideration.

This longitudinal cohort-based study has relevant strengths and weaknesses. The study is rooted in validated sequencing methods (SGS) (92, 93) and modeling (star-like phylogeny) (63) that allow inference of the TF virus in AAi and escape of early anAbs in EAi. Limitations include a relatively homogeneous study population of all men, mostly MSM from a single US city, and interindividual variability in adherence to study visits and ART. However, given the frequency of our study visits (monthly, as well as an additional week 2 visit) and the close relationship with the San Francisco Department of Public Health’s Getting to Zero program (94), aimed at identifying newly diagnosed individuals with HIV, providing them with immediate ART, and closely following them, our well-characterized cohort was able to capture the majority of these data. Further studies in other populations, including women from diverse regions, are needed to understand the generalizability of findings. Studying virus neutralization also has important caveats. In vitro assays for neutralization potency do not directly represent in vivo activity, but are validated correlates of relative neutralization activity that can be compared across studies and cohorts. Here, we assess neutralization potency on ART with extracted IgG, which is a frequently used but imperfect correlate of the TZM.bl assays using the standardized plasma dilutions. As the field works to develop other methods, including ART-resistant backbones and distinct viral vectors (17, 51), these assays will become more comparable across studies.

In summary, we found that ARTi in acute HIV prevented subsequent formation of anAbs, unless the participant experienced additional antigen in the form of rebound viremia. ARTi in early HIV, in contrast, enabled development of anAbs that continued to expand in breadth and potency over time on suppressive ART. Given the relative lack of virus diversity in the HIV reservoir after acute and early ARTi, these primed or continuously maturing anAb responses represent an attractive target for therapeutic vaccination with the goal of increasing the breadth and potency of responses to elicit durable virus suppression.

## Methods

### Sex as a biological variable.

Our study included 23 participants, all of whom reported being of male sex and male gender. The study population was drawn from a clinical cohort that is almost entirely male. This limits the generalizability of the results. Future work in women is needed to confirm findings.

### Participants.

Individuals with newly diagnosed acute/early HIV infection gave consent and were enrolled in the UCSF Treat Acute HIV study from December 1, 2015, to November 30, 2020. Participants were provided with immediate ART (tenofovir/emtricitabine plus dolutegravir), linked to clinical care, and followed monthly for 24 weeks and then approximately every 3 months thereafter. For the current study, a total of 23 participants were included for whom (a) viremic plasma at ART initiation was available and of adequate quality for SGS, (b) sufficient archived clinical laboratory testing was available to estimate date of detectable HIV infection, and (c) 2 or more post-ARTi plasma sample time points were available. At each visit, detailed interviews were performed, including questions regarding current medications, medication adherence, intercurrent illnesses, and hospitalizations. In addition, peripheral blood sampling at each visit was performed to measure plasma HIV RNA, CD4^+^ T cell count, and clinical labs, as well as blood for storage.

### Sample preparation, viral load measurements, and time to ARTi (EDDI).

Whole-blood samples were collected in EDTA and acid citrate dextrose tubes and processed within 24 hours. PBMCs and plasma were separated using Ficoll gradient purification and stored at –80°C. Viral loads were measured by commercially available clinical viral load assays (Abbott Real Time PCR assay, limit of detection <40 copies/mL). Timing of ARTi was calculated using the Infection Dating Tool (https://tools.incidence-estimation.org/idt/), which included the EDDI, along with a “confidence interval” for early probable and late probable date based on each participant’s prior clinical HIV test results, as well as the baseline study results (52, 53).

### Gp120 binding antibodies.

Gp120 binding antibodies were assessed by indirect qualitative ELISA as previously described (6). In brief, 96-well plates were coated with 2 μg/mL recombinant YU2c gp120 protein (Immune Technology, catalog IT-001-0027p) overnight and incubated with 100 μL of heat-inactivated participant plasma in 5-fold dilutions (ranging 1:20 to 1:312,500) as primary antibody, then 100 μL HRP-conjugated secondary antibody (Jackson ImmunoResearch, catalog 109-035-098) at 1:10,000 dilution. Blocking and dilutions were performed in B3T blocking buffer (150 mM NaCl, 50 mM Tris-HCl, 1 mM EDTA, 3.3% FBS, 2% bovine albumin, 0.07% Tween 20). After antibody incubations, 100 μL KPL Sureblue TMB peroxidase substrate (SeraCare) was added and incubated at room temperature for 10 minutes, after which 100 μL 1 N sulfuric acid was added and absorbance at 450 nm was read (H4 synergy plate reader, BioTek). Two or more replicates were performed for each sample. HIV-negative donor plasma was analyzed in parallel as negative control. Absorbance values were plotted as a function of antibody concentration, and nonlinear curve fit by sigmoidal dose-response (variable slope) model with area under the curve (AUC) measurement was computed in Prism (GraphPad Prism version 10.0.2). Assay background was calculated as 3 standard deviations above the mean AUC of HIV-negative donor plasma.

### IgG purification.

Plasma was heat-inactivated at 56°C for 1 hour. Then, IgG was extracted using the Protein G Gravitrap system (Cytiva) according to manufacturer protocol. Buffer exchange of purified IgGs was then performed by 3 PBS washes in Amicon Ultra-4 filters with 30 kDa cutoff (MilliporeSigma). IgG was then sterile-filtered in 0.22 μM centrifugal filter tubes (Corning Spin-X). IgG concentrations were quantified by absorbance throughput at 280 nm on a Synergy H4 plate reader (BioTek).

### SGS.

Plasma virus from day of ARTi was characterized by SGS for all participants. Plasma virus at post-ARTi time points was additionally sequenced for participants with viremia after ARTi (participants 8028, 8043, and 8016). SGS was performed per previously described methods (73, 89). Briefly, plasma samples were thawed on ice, and viral RNA extraction was performed on a volume of plasma estimated to contain about 20,000 virions per clinical viral load measurement using EZ virus Mini Kit 2.0 (QIAGEN) per manufacturer protocol. cDNA template was synthesized by Superscript III system (Life Technologies) using R3B3R antisense primer (5′-ACTACTTGAAGCACTCAAGGCAAGCTTTATTG-3′) (93), and Env region was amplified by nested PCR using high-fidelity Platinum Taq DNA polymerase (Life Technologies) in 96-well plates under previously described conditions (73, 89). Input DNA was titrated to yield positive amplicons in less than 30% of reactions, reducing polymerase-induced errors (92, 93). Reactions containing approximately 3 kb amplicons were sequenced as below.

### Sequence analysis and identification of TF/early virus.

SGS amplicons were sequenced by MiSeq platform (Illumina). Sequence analysis was performed in Geneious Prime software (v2023.2.1). Raw sequencing reads were aligned to HXB2 (GenBank accession K03455.1) or other clade B consensus sequence. Resultant contigs were inspected for adequate coverage, and consensus sequences with large deletions or ambiguous bases at 75% identity were discarded. For participants with few sequences (8011, 8063, 8038), consensus sequences with ambiguous bases at 75% identity were reanalyzed at 50% identity. For sequences containing few (1 or 2) positions with ambiguous bases as a result of poor sequencing coverage (not “double peaks”), ambiguous bases were manually edited to agree with the consensus base at that position for that participant. Consensus sequences of full-length Env gp160 were aligned by MUSCLE algorithm (95) for each participant. Maximum-likelihood nucleotide trees were compiled by PhyML (http://atgc.lirmm.fr/phyml/) with 100 bootstrap replicates and optimized to topology, branch length, and substitution rate.

Diversity and multivariant infection were assessed subjectively by visual inspection of nucleotide phylogenetic tree and highlighter plots (Los Alamos National Laboratory online Highlighter tool) (92) and objectively by fitting of alignments to random viral evolution models using the Los Alamos National Laboratory online Poisson Fitter tool (63). Populations exhibiting SLP or near-SLP by either method were presumed to have single TF transmission. For participants with plasma virus populations not conforming to SLP, the presence of MVT was determined by inspection of phylogenetic trees and highlighter plots for distinct clades with conserved nucleotide motifs.

Amino acid translations of gp160 sequences were also aligned by MUSCLE algorithm, and phylogenetic trees were assembled by the same methods as for nucleotide trees using Geneious Prime software. Sequences with frameshift or nonsense mutations leading to premature stop codons were discarded from amino acid alignments.

### TF/early virus (Env) selection and cloning.

In participants whose plasma virus populations conformed to SLP, as was the case for AAi participants with single-variant transmission, TF virus was inferred as the consensus of the nucleotide sequence alignment (92). In AAi participants with MVT, we inferred TF viruses as the most recent common ancestors of each individual clade. In EAi participants, all of whom defied SLP, TF could not be inferred, and therefore we identified “early” virus sequences that were representative of the virus population present at ARTi. In selecting such early viruses, we inspected amino acid highlighters and chose sequences in which the Env regions generally targeted by early anAb responses, namely gp120 variable regions (V1–5) and potential N-linked glycosylation sites, were representative of the individual’s broader virus population. For EAi participants with MVT, we selected an “early” virus from each distinct clade.

Selected TF and early viruses were molecularly cloned by amplification of SGS product and insertion into pcDNA 3.1 Zeo(+) expression vector (Invitrogen, Thermo Fisher Scientific) by Gibson assembly. For each participant, bespoke Gibson assembly primers were generated by NEBuilder online tool (New England Biolabs, https://nebuilder.neb.com). Some Env plasmids, including tier 1 viruses MN.3 (HM215430.1) and SF162 (EU123924.1), were synthesized and cloned by Twist Bioscience; Envs from participants 8010, 8011, 8026, 8061, and 8014 were also synthesized and cloned by Twist Biosciences. All cloned Envs were then sequenced to confirm the identity of the original sequence.

Cloned plasmids were transformed into STBL2 competent cells (Thermo Fisher Scientific) and grown on antibiotic-containing Luria broth agar plates for 48 hours at room temperature. After incubation, single colonies were selected and grown overnight in liquid broth at 30°C. Plasmid DNA was isolated using QIAprep Spin Miniprep Columns (QIAGEN). Correct insertion of desired Env insert was confirmed by sequencing (MiSeq, Illumina).

### Pseudovirus generation and titration of infectivity.

Pseudovirus was prepared as described previously (89) by cotransfection of Env-containing plasmid with SG3ΔEnv plasmid (obtained through the NIH HIV Reagent Program, Division of AIDS, National Institute of Allergy and Infectious Diseases [NIAID], NIH: Human Immunodeficiency Virus Type 1 [HIV-1] SG3ΔEnv Non-infectious Molecular Clone, ARP-11051, contributed by John C. Kappes and Xiaoyun Wu (University of Alabama at Birmingham, Birmingham, Alabama, USA) in HEK293T cells (ATCC) by FuGENE 4K transfection system (Promega). Culture supernatants were harvested 48–72 hours after transfection. Cellular debris was removed through 0.4 μm filtration, and resultant viral stocks were stored at –80°C. Multiplicity of infection (MOI) of pseudovirus stocks was determined by TZM.bl β-galactosidase assay as previously described (89).

### Neutralization assays.

Virus neutralization by purified plasma IgG was assessed in TZM.bl assay as described previously (73, 89). In brief, 10,000 TZM.bl cells (obtained through the NIH HIV Reagent Program, Division of AIDS, NIAID, NIH: TZM.bl Cells, ARP-8129, contributed by John C. Kappes, Xiaoyun Wu, and Tranzyme Inc.) per well in 96-well plates were incubated for 48 hours in the presence of purified participant plasma IgG in 5-fold dilutions ranging 1,000 to 0.0128 μg/mL, 40 μg/mL DEAE-dextran, and 3,333 infectious units of autologous pseudovirus (MOI of 0.3). Assays were performed in duplicate with challenge of antibody against pseudotyped murine leukemia virus in parallel as negative control. After 48-hour incubation, luciferase assay was performed using the Firefly Luciferase assay system (Promega) per manufacturer instructions. Luminescence in relative light units (RLUs) was quantified using an H4 Synergy plate reader (BioTek). RLUs were plotted as a function of antibody concentration to generate dose-response curves (GraphPad Prism version 10.0.2). The antibody concentration that neutralized 50% (IC_50_) of pseudoviral infection was calculated by 4-parameter logistic regression fit. Tier 2 heterologous panel of pseudoviruses and infectious molecular clones was provided by the laboratory of George Shaw (University of Pennsylvania, Philadelphia, Pennsylvania, USA).

### Statistics.

Statistical analyses were performed in GraphPad Prism (version 10.0.2) as outlined in the figure legends, and 2-tailed, nonparametric tests were used for all analyses unless otherwise specified. *P* < 0.05 was considered significant.

### Study approval.

All participants provided written informed consent, and the institutional review boards of the UCSF and the University of Pennsylvania approved the research.

### Data availability.

Sequence data were submitted to GenBank (accession OR922877–OR923336). See the Supporting Data Values file for numerical data underlying figures and means.

## Author contributions

SAL, SD, RH, HH, VP, SS, and SGD managed the Treat Acute HIV cohort, collected clinical data, and coordinated sample collection and processing. GDW, JJ, FEM, RK, SM, HC, KP, JC, and KJB performed laboratory experiments. GDW, RML, FEM, RK, and KJB analyzed data. GDW and KJB wrote the manuscript; all authors reviewed and edited the manuscript.

## Supplementary Material

Supplemental data

ICMJE disclosure forms

Supporting data values

## Figures and Tables

**Table 2:**
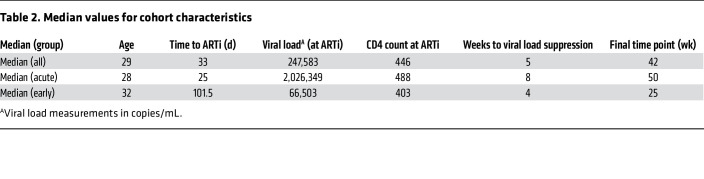
Median values for cohort characteristics

**Table 1:**
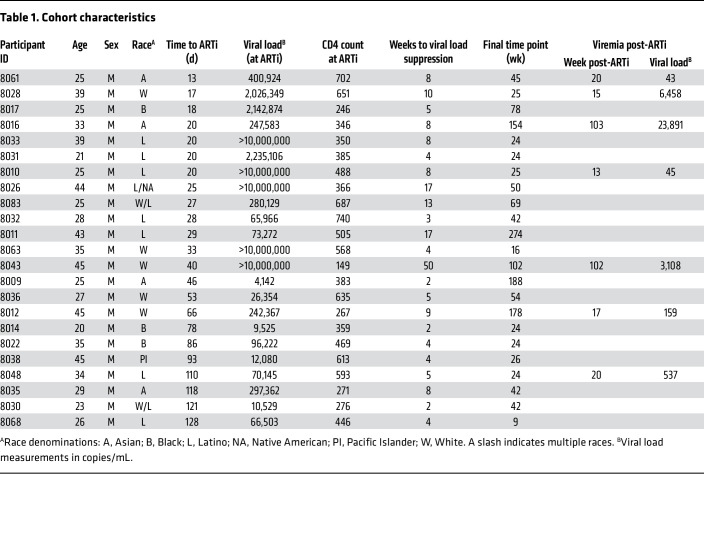
Cohort characteristics

## Version 1 04/23/2024

In-Press Preview

## Version 2 06/03/2024

Electronic publication
