# Supplementary material for: Autologous neutralizing antibody responses after antiretroviral therapy in acute and early HIV-1
Source: J Clin Invest. 2024 Apr 23;134(11):e176673. doi: 10.1172/JCI176673 (PMC11142743; doi:10.1172/JCI176673)
Supplement: Supplemental data [file jci-134-176673-s032.pdf]

Figure S1

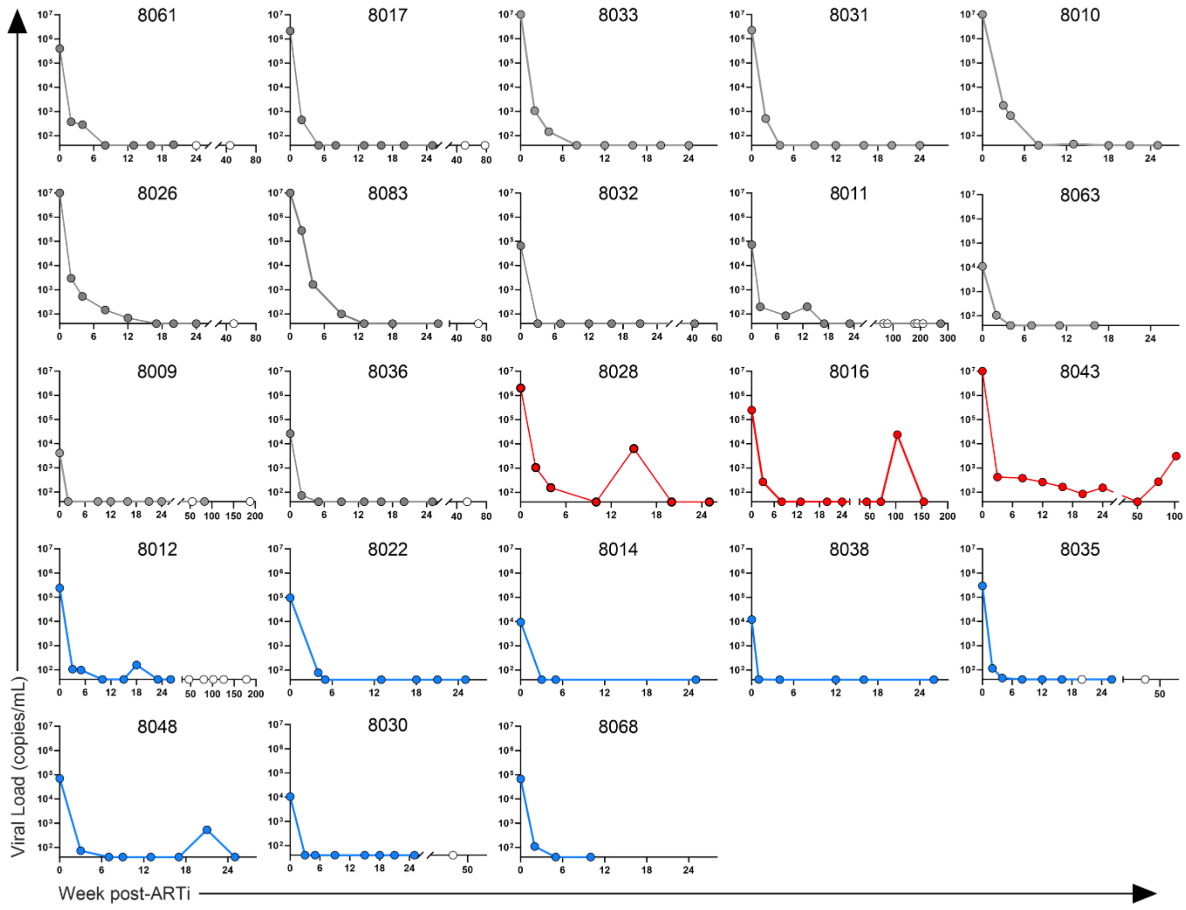

Figure S1: Viral Load measurements of individual participants

Longitudinal viral load measurements (in copies/mL) for all participants. AAi without rebound in gray, AAi with rebound in red, and EAi in blue. White circles denote timepoints where plasma is available but viral loads were not measured.

Figure S2

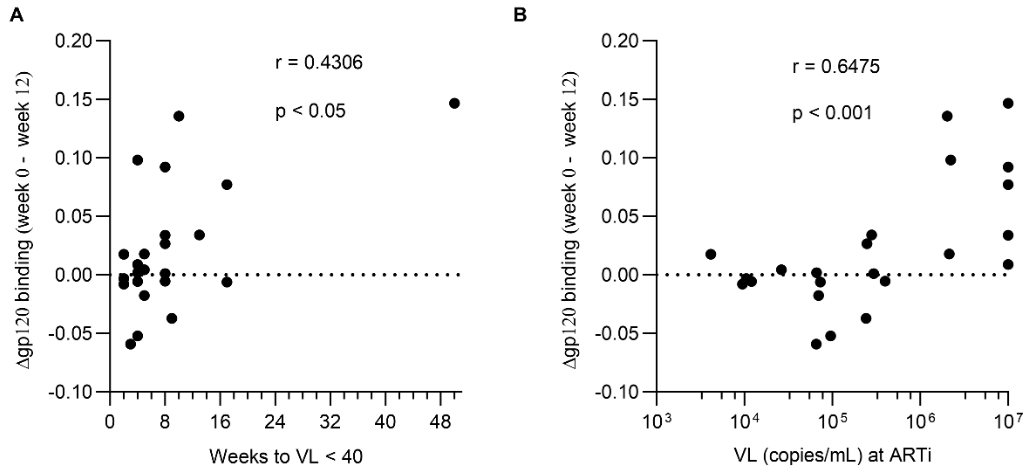

Figure S2: Change in binding antibodies in first 12 weeks of ART is associated with markers of acute infection.

Change in binding antibody levels between week 0 and week 12 plasma as measured by area under the curve from ELISA binding assay correlates positively with (**A**) weeks to first undetectable viral load and (B) viral load at ARTi, both markers of acute infection. Spearman R values and p values are presented.

Figure S3

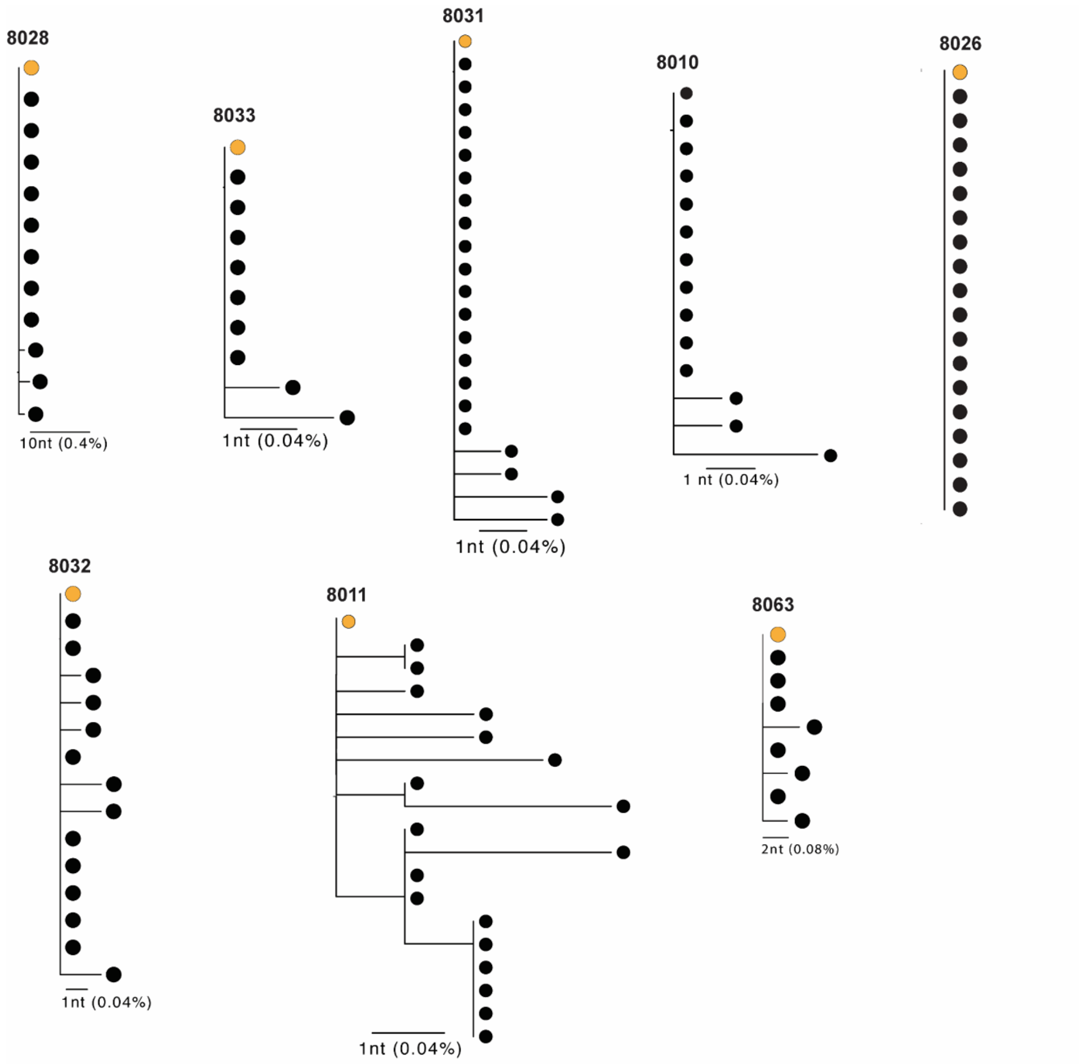

Figure S3: Phylogenies of single Transmitted Founder AAi participants

Nucleotide maximum-likelihood phylogenies of Acute ARTi (AAi, <60 days to ARTi) participants identified as possessing single transmitted/ founder virus. Each solid black node represents a gp160 Env sequence obtained by SGS, while solid orange nodes represent sequence cloned for phenotypic testing. For Participant 8010, no sequence was successfully cloned, this this participant was absent from autologous neutralizing antibody assessments.

Figure S4

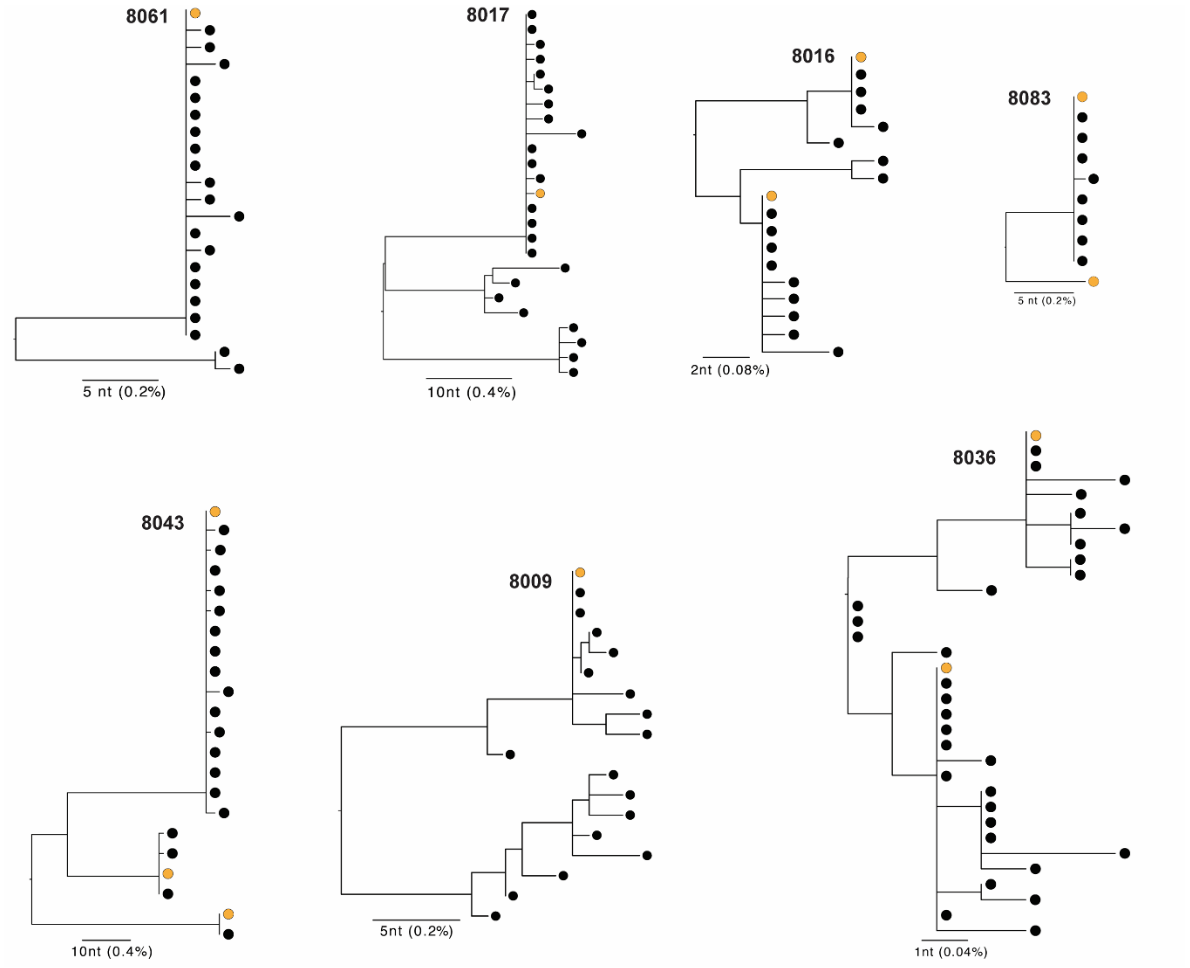

Figure S4: Phylogenies of Multiple Transmitted Founder (MVT) AAi participants

Nucleotide maximum-likelihood phylogenies of Acute ARTi (AAi, <60 days to ARTi) participants identified as possessing multiple transmitted/ founder virus (multivariant transmission, MVT). Each solid black node represents a gp160 Env sequence obtained by SGS, while solid orange nodes represent sequences cloned for phenotypic testing.

Figure S5

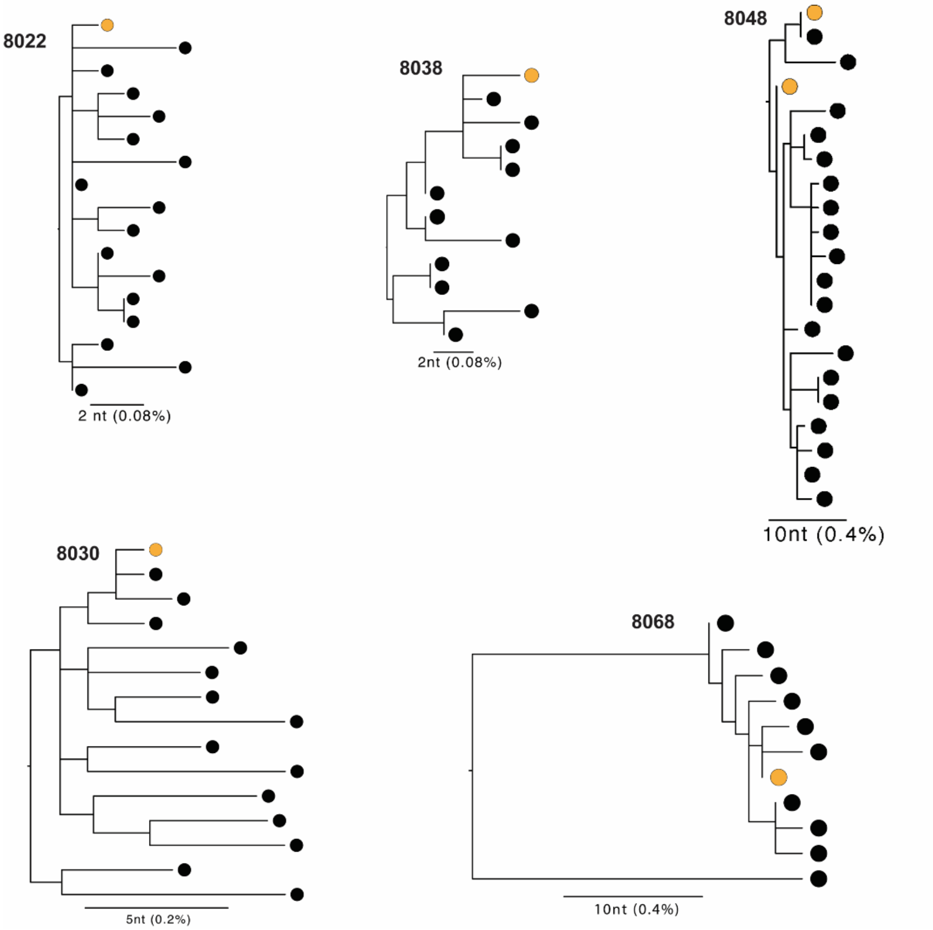

Figure S5: Phylogenies of single Transmitted Founder EAi participants

Nucleotide maximum-likelihood phylogenies of Early ARTi (EAi, >60 days to ARTi) participants identified as possessing single transmitted/ founder virus. Each solid black node represents a gp160 Env sequence obtained by SGS, while solid orange nodes represent sequences cloned for phenotypic testing.

Figure S6

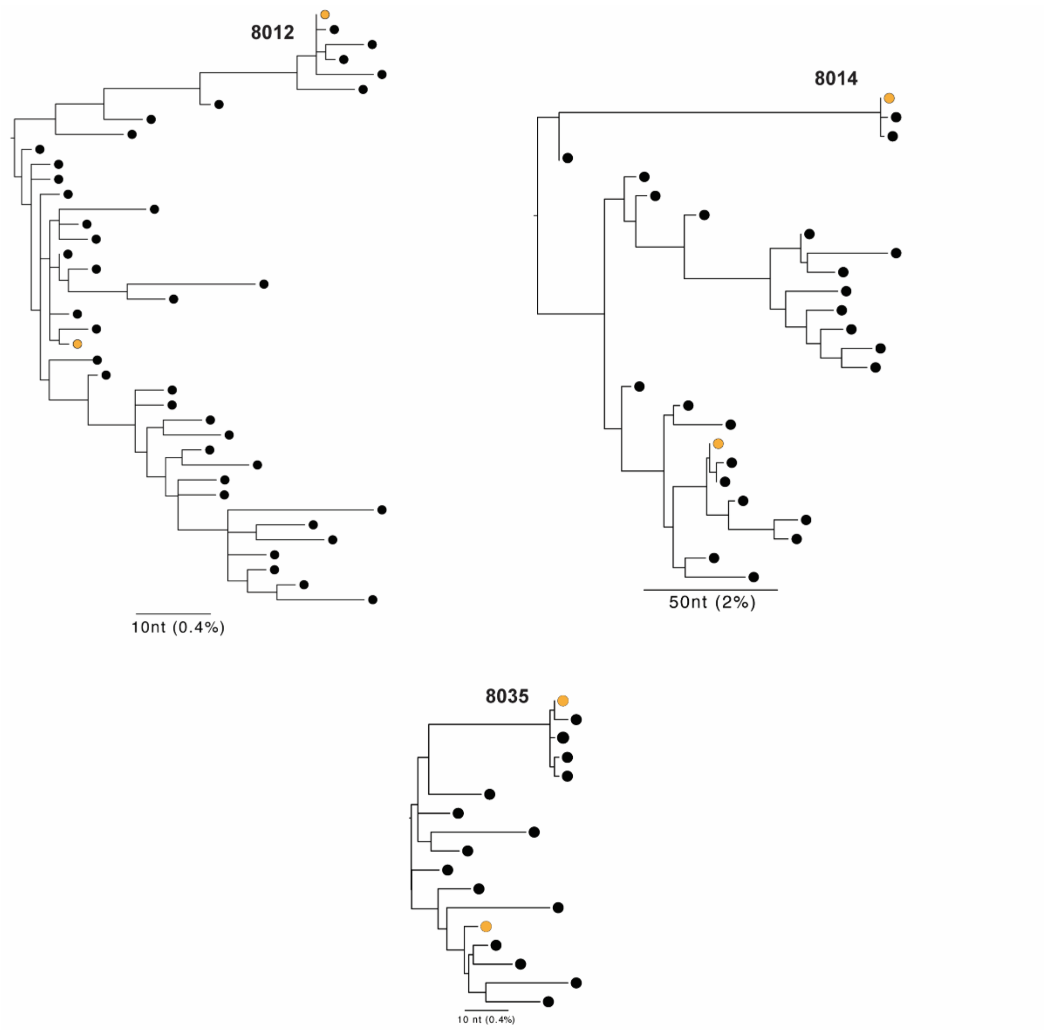

Figure S6: Phylogenies of Multiple Transmitted Founder (MVT) EAi participants

Nucleotide maximum-likelihood phylogenies of Early ARTi (EAi, >60 days to ARTi) participants identified as possessing multiple transmitted/ founder virus (multivariant transmission, MVT). Each solid black node represents a gp160 Env sequence obtained by SGS, while solid orange nodes represent sequences cloned for phenotypic testing.

Figure S7

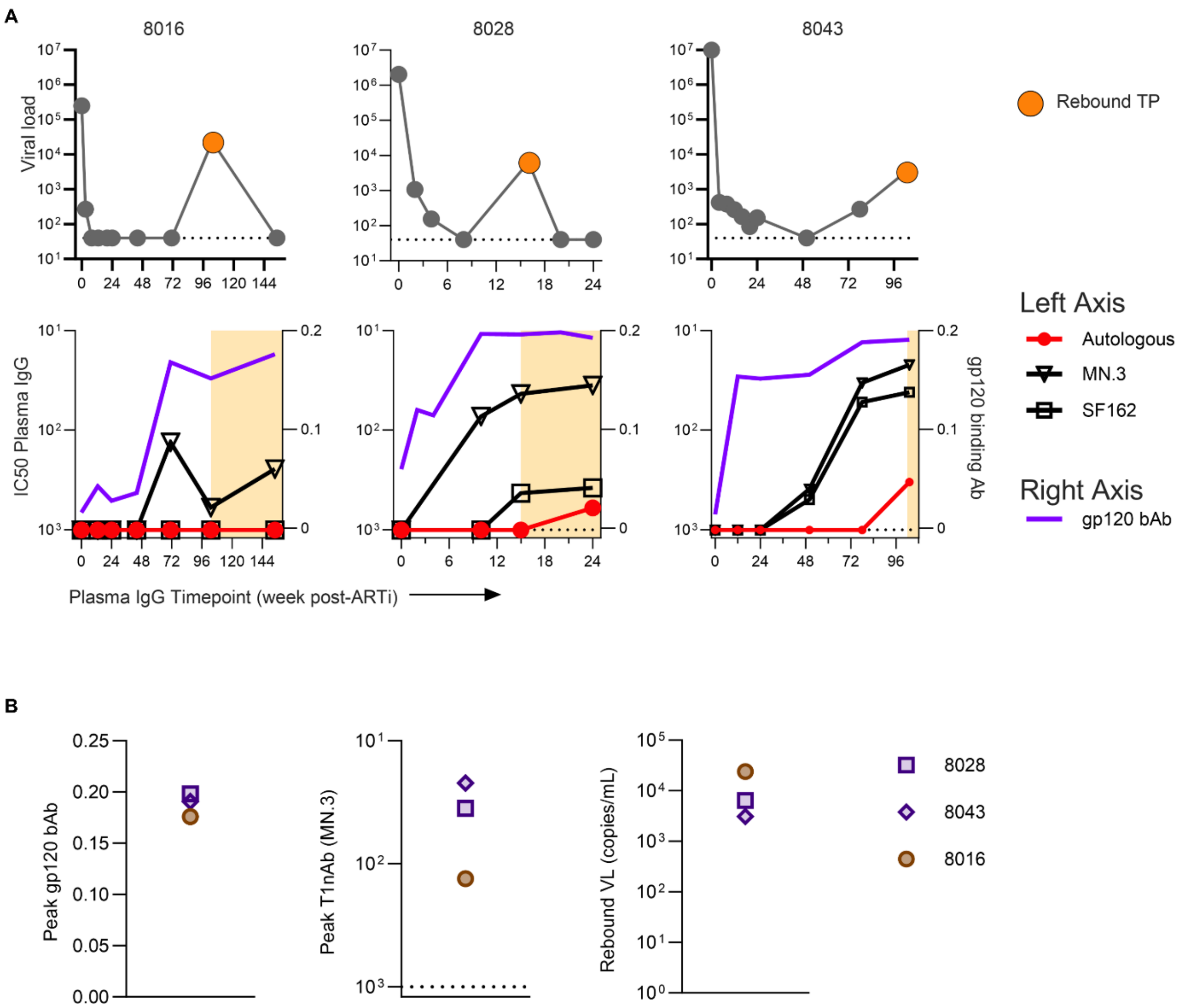

Figure S7: Viral load and antibody kinetics in AAi with rebound participants

In panel (**A**), viral load (grey) is plotted over time with rebound timepoint denoted in orange in top graph, while bottom graph shows autologous neutralizing antibody IC50 (red, left axis), Tier 1 neutralizing antibody (black, left axis), and gp120 binding antibody level measured as ELISA area under the curve (purple, right axis). Shaded orange area represents timepoints after rebound episode. In panel (**B**), the peak gp120 binding antibody (left), peak Tier 1 neutralizing antibody (middle)), and rebound viral load (right) of each participant are plotted.

Figure S8

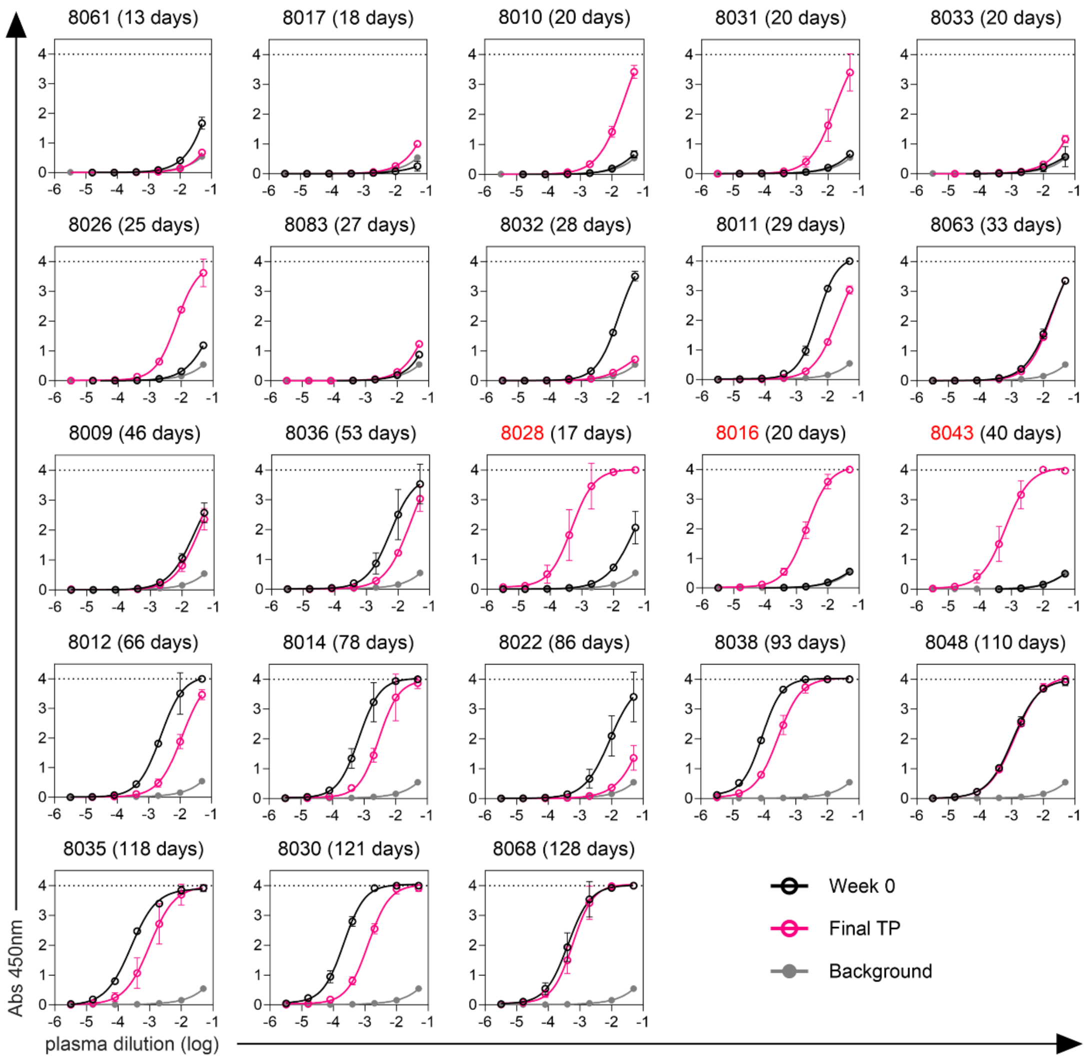

Figure S8: gp120 binding activity in plasma at ARTi and final timepoint

ELISA curves for gp120 binding assays with absorbance reading at 450nm plotted against plasma dilution for week 0 (black, ART initiation timepoint) and final timepoint (pink) are plotted for each participant. Data from 2-4 replicate experiments are plotted as mean value with SEM. Background (grey) is 3 standard deviations above mean value of assay run with HIV-negative healthy donor plasma.
